# 474. Differences In Clinical Manifestation, Prognostic Factors, and Outcomes Between Patients With Community-onset And Nosocomial Candidemia

**DOI:** 10.1093/ofid/ofac492.532

**Published:** 2022-12-15

**Authors:** Yung-chun Chen, Chao-chin Chang

**Affiliations:** Department of Critical Care Medicine, Taichung Veterans General Hospital, Taichung, Taichung, Taichung, Taiwan; Graduate Institute of Microbiology and Public Health, National Chung Hsing university, Taichung, Taichung, Taiwan

## Abstract

**Background:**

Candidemia is an invasive and deadly infectious disease. The associated risk factors of candidemia include immunosuppression, broad-spectrum antibiotics, catheters, glucocorticoids, critical illness, surgery, neonates, previous candida colonization, and illicit drug use. In previous studies, the characteristic of community-onset candidemia was distinct from nosocomial candidemia.

**Methods:**

We selected patients who were admitted for equal or more than two days in Taichung Veterans General Hospital of Taiwan from 2015 to 2018 with electronic medical charts. The diagnosis of candidemia was defined as *Candida* species identified in blood cultures by commercial identification systems. We applied Kaplan-Meier survival curves with a log-rank test and built multivariate Cox proportional-hazards models for risk factors associated with in-hospital mortality. The purpose of this study is to investigate the difference in the characteristics, outcomes, and associated risks of death between community-onset and nosocomial candidemia.

**Results:**

A total of 339 patients were included for analysis. The incidence was 1.50 per 1000 admission person-years. 64.60% of patients were male, and the mean age was 64.24-year-old. The characteristics of patients were similar in both groups except for the status of port-A, glucocorticoid usage, and diabetes mellitus. In the community-onset group, a higher proportion of *C. glabrata* and *C. parapsilosis,* but fewer *C. albicans* and *C. tropicalis* were identified than in the nosocomial group. The all-cause mortality rate was 55.75%. Multivariate Cox proportional-hazards model showed that nosocomial (hazard ratio; HR, 1.96; 95CI, 1.291 - 2.97 ; *p* =0.002), glucocorticoids within seven days before candidemia (HR, 1.68; 95CI, 1.21 -2.32, *p* = 0.002), and septic shock (HR, 1.85; 95CI, 1.292 - 2.641, *p* < 0.001) were associated with poor outcome whereas treatment with an echinocandin within two days after candidemia was a protective factor (HR, 0.65; 95CI, 0.466 - 0.911, *p* = 0.012).

Survival curves analysis between community-onset candidemia and nosocomial candidemia

**Figure:**
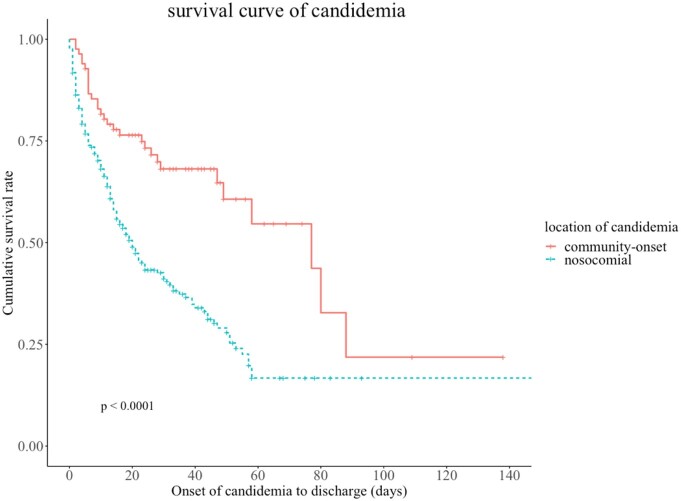

A log-rank test was applied to assess the statistical significance, and a p-value less than 0.05 was considered statistically significant.

**Conclusion:**

In conclusion, candidemia is a lethal disease with a better outcome noted in those with community-onset candidemia. Species diversity existed in both groups, and Early initiation of echinocandin therapy within two days could reduce 35% mortality.

**Disclosures:**

**All Authors**: No reported disclosures.

